# Atlanta Medical College

**Published:** 1892-05

**Authors:** 


### *CLINICAL REPORT.*

### ATLANTA MEDICAL COLLEGE.

[Reported from the Medical Clinic by H. W. Terrell, class 1892.]

October 30, 1892, J. B. S., married; white, age 31; residence, Alabama; occupation, farmer; history, married about eight years; was in good health up to seven years ago. Sickness began with severe pain in stomach while ploughing in spring of year. Pain came on suddenly. First attack lasted about three hours. Did not vomit. Took dose of assafœtida, got easy, then returned to ploughing; soon had to stop, did not go to work in one month or six weeks. Then had a second attack which lasted two or three hours. Got entirely over that. In about one month had third attack about same course. Weight before taken sick was about 160 pounds; strong and healthy. Never had any venereal trouble. Attacks get more severe each time, pain most severe in stomach. Attacks are getting more frequent and worse. Appetite generally good, sometimes vomit after a meal, though may not vomit until about night. Present weight 110 pounds. Bowels are very costive and have been for some time. Have to use enema of warm water to move them. Has been doing this for two years. Stools "hard and round balls." Do not pass much urine. Last night vomited about three quarts hot fluid.

Upon examination find urine normal, fluid and gas in abdomen.

### DIAGNOSIS—CIRRHOSIS OF LIVER.

Was given following treatment:

℞. Pv. ipecac, gr. ij.
Ext. nux. vom., gr. v.
Aloin, gr. viij.

M. ft. Pil. No. xx. Sig.—One once or twice a day.

℞. Tr. nux. vom., ʒiv.

Dil. mur. acid, ʒiv.

Pepsin (lac.) ʒij.

Camph. water, ℥ijss.

M. Sig.—Teaspoonful in wine glass water after each meal.

30. R. Quinine,
Sul. iron, aa. gr. xxx.
Arsenic, gr. iss.
Strychnine, ⅔ gr.
Ext. gentian, gr. xv.

M. ft. Pil. No. xxx. Sig.—One after each meal, from one-half to hour after other is taken.

November 10, 1891, J. M. D., age 47; white; occupation, painter. Has been a painter for eighteen years. Father and mother both dead. Father died with inflammation of bowels. Mother died from "old age." One brother died with scarlet fever when young. Patient has been bothered with rheumatism about seven years. Pain most severe in leg. First attack began in thigh and pain lasted about ten or fifteen minutes; often the pain lasts all night. Pain is not confined to thigh; attacked knee and foot last year. Knees bother all the time, has no use of them. When walking he staggers as if drunk. Has been troubled this way for two years. Flesh feels "dry and dead" in legs. Of late has not been able to work. Has had "cramp colic" three or four times. Has had "painter's colic" three times. Last attack of "painter's colic" about two years ago, from first to second attack about six or seven years. Has no pain except in legs which extends to hypogastric region. Patient worse than one year ago. Patient says he had syphilis about ten years ago, but was troubled with it only three weeks. Pains very severe, lancinating.

DIAGNOSIS—LOCOMOTOR ATAXIA.

Treatment:

R. Iod. pot., ℥iss.

Bichlor. merc., gr. iss.

Tr. gentian, ℥ij.

Water, ℥iv.

M. Sig.—Teaspoonful in one-half glass water after meals.

